# Metabolic Profiles, Bioactive Compounds, and Antioxidant Capacity in *Lentinula edodes* Cultivated on Log versus Sawdust Substrates

**DOI:** 10.3390/biom11111654

**Published:** 2021-11-08

**Authors:** Miso Nam, Ji Yeon Choi, Min-Sun Kim

**Affiliations:** Food Analysis Research Center, Korea Food Research Institute, Wanju 55365, Korea; msnam@kfri.re.kr (M.N.); jychoi@kfri.re.kr (J.Y.C.)

**Keywords:** *Lentinula edodes*, metabolite profiling, bioactive compounds, antioxidant capacity, log bed cultivation, sawdust media cultivation

## Abstract

*Lentinula edodes* (shiitake) is a popular nutritious edible mushroom with a desirable aroma and flavor. Traditional cultivation of *L. edodes* on beds of logs has been replaced by cultivation on sawdust, but the effects of cultivation changes on *L. edodes* mushrooms have not been well characterized. We determined the metabolic profile, bioactive compounds, and antioxidant capacity in *L. edodes* grown on log or sawdust substrates. Metabolic profiles of *L. edodes* extracts were determined by ^1^H nuclear magnetic resonance (NMR) and ultra-performance liquid chromatography to quadrupole time-of-flight mass spectrometry. Principal component analysis score plots from ^1^H NMR analysis showed clear differences between samples. Concentrations of primary metabolites, especially amino acids, generally decreased in *L. edodes* grown on logs compared to sawdust. Phenolic compounds showed variations in concentration depending on the cultivation method. Bioactive compounds and their antioxidant capacity were analyzed spectrophotometrically. *L. edodes* cultivated on logs had high concentrations of bioactive compounds with strong antioxidant capacity compared to *L. edodes* cultivated on sawdust. Thus, the concentration of primary metabolites was high in *L. edodes* grown on sawdust, which produces a high growth rate. In contrast, log-cultivated *L. edodes*, which were similar to wild mushrooms, had high levels of bioactive compounds and high antioxidant capacity. This information is useful for determining optimal cultivation conditions for nutritional and medicinal uses of *L. edodes* mushrooms.

## 1. Introduction

The shiitake mushroom, *Lentinula edodes*, is nutritious and has a unique flavor that makes it the second most popular edible medicinal mushroom in the world [1]. It is widely cultivated in many Asian countries [2]. Production of *L. edodes* has increased faster than any other mushroom, accounting for about 25% of global edible fungus production [3,4]. *L. edodes* is valuable as food and medicine, rich in essential amino acids, dietary fiber, vitamins, and minerals but low in calories and fat [5,6,7,8,9]. Its bioactive compounds, including polysaccharides, terpenoids, steroids, phenols, nucleotides, and glycoprotein derivatives [10], may reduce the risk of hypertension, hypercholesterolemia, diabetes mellitus, and cancer [10,11].

*L. edodes* is manufactured under conditions similar to its natural environment on either synthetic sawdust substrates or hardwood logs, such as oak. Sawdust cultivation, which accounts for about 80% of the cultivation on substrates [12], reduces the cultivation period to a few months compared to two years using logs [13]. However, mushrooms grown on sawdust are considered inferior in flavor and nutrition to those cultivated on logs. Therefore, it is important to compare the biochemical composition and therapeutic effects of *L. edodes* grown on different substrates. While the proximate composition, free amino acids, and 5′-GMP concentrations for *L. edodes* cultivated on logs versus sawdust have been determined [13], the broader biochemical composition and biological activities of *L. edodes* mushrooms cultivated under different conditions have not been determined.

Here, we determined the metabolic profile, bioactive compounds, and antioxidant capacity of *L. edodes* cultivated on logs versus sawdust. The complementary use of nuclear magnetic resonance (NMR) and mass spectrometry (MS) provides excellent metabolic profiling by balancing the strengths and limitations inherent in each technique. We analyzed the most abundant primary metabolites of *L. edodes* extracts using ^1^H NMR and the secondary metabolites using ultra-performance liquid chromatography to quadrupole time-of-flight mass spectrometry (UPLC-QTOF-MS). The bioactive compounds and their antioxidant capacity were determined spectrophotometrically. We characterized the differences in metabolites and estimated the bioactive compound and antioxidant capacity of *L. edodes* based on different cultivation conditions.

## 2. Materials and Methods

### 2.1. Standards and Reagents

Deuterated methanol (99.8 atom % deuterium) and deuterium oxide (99.9 atom % deuterium) were obtained from Cambridge Isotope Laboratories, Inc. (Andover, MA, USA). 3-(trimethylsilyl)propionic-2,2,3,3-d4 acid (TSP), Folin-Ciocalteu’s phenol reagent, sodium carbonate, diethylene glycol, gallic acid, rutin, catechin, 2,2-diphenyl-1-picrylhydrazyl (DPPH), L-ascorbic acid, formic acid and hydrochloric acid were obtained from Sigma-Aldrich (St. Louis, MO, USA), and sodium hydroxide was purchased from Junsei Chemical (Tokyo, Japan). Methanol, water, and acetonitrile were of the highest commercial grade and obtained from Honeywell (St. Muskegon, MI, USA). All chemicals were of analytical grade.

### 2.2. Mushroom Material

*L. edodes* mushrooms were obtained commercially about 2 kg per each sample from local markets in South Korea. The *L. edodes* mushrooms were collected between April and May 2020. A total of 28 samples for *L. edodes* cultivated on log bed and 53 samples for *L. edodes* cultivated on sawdust media were collected. Detailed collection strains information is provided in Table S1. Fruiting bodies of mushrooms were chopped and freeze-dried as soon as they were collected. Dried fruiting bodies were ground to a fine powder and stored at −80 °C until the experiment.

### 2.3. Metabolite Profiling Using ^1^H NMR Analysis

500 μL of methanol-d_4_, 400 μL of 0.2 M sodium phosphate buffer in D_2_O (pH 7.0 ± 0.1), and 100 μL of 6 mM TSP were added to approximately 50 mg of dried powder as extraction solvents. D_2_O was used as the internal lock signal and TSP was used as an internal standard with a chemical shift (δ) of 0.0 ppm. The mixtures were sonicated for 20 min and adjusted to pH 7.0 ± 0.1 using 0.2 M NaOH and 0.2 M HCl solutions. After centrifugation (12,000 rpm, 20 min, room temperature), the supernatants were transferred to 5 mm (ø) NMR tubes for NMR analysis. ^1^H NMR spectra were acquired on a Bruker Avance III HD 800 MHz FT-NMR Spectrometer (Bruker BioSpin Co., Billerica, MA, USA) at 298 K using a 5-mm triple-resonance inverse cryoprobe with Z-gradients. The 1D NOESY pulse sequence was applied to suppress the residual water signal. For each sample, 64 transients were collected into 64,000 data points using a spectral width of 16,393.4 with a relaxation delay of 2 s and an acquisition time of 2 s. A 0.5 Hz line-broadening function was applied to all spectra for Fourier transformation, followed by phasing and baseline correction. Signal assignments for representative samples were achieved using 2D total correlation spectroscopy, 800 MHz NMR database of Chenomx NMR Suite Version 8.6 (Edmonton, AB, Canada), and spiking experiments. The metabolites were quantified using the Chenomx NMR Suit Version 8.6.

### 2.4. UPLC-QTOF-MS Analysis

The acquisition was performed using an Exion LC™ AD system coupled to a X500R QTOF system (all devices from AB SCIEX, Concord, ON, Canada), equipped with an electrospray ionization (ESI) source. The chromatographic separation analysis was carried out with an Acquity UPLC HSS T3 column (2.1 mm × 100 mm, 1.7 μm; Waters) at 40 °C; a binary gradient separation was performed at a flow rate of 0.45 mL/min. Mobile phase A consisted of 0.1% formic acid in water and mobile phase B consisted of 0.1% formic acid in acetonitrile. The linear gradient elution was as follows: 0–0.7 min, 1–5% B; 0.7–2.7 min, 5–25% B; 2.7–4.5 min, 25–35% B; 1 min, isocratic 35% B; 5.5–6.5 min, 35–45% B; 1 min, isocratic 45% B; 7.5–8.5 min, 45–60% B; 8.5–9.0 min, 60–100% B; and 9.0–11 min (post-acquisition time), 1% B to re-equilibrate the column. The total run-time for each injection was 13 min and the injection volume was 5 μL. The mass spectrometer was operated in positive and negative ionization modes and acquired data in the mass range from 50 to 1100 *m*/*z*. The following parameter settings were used: ion spray voltage: 5500 V, temperature: 500 °C, curtain gas: 30 psi, declustering potential: 90 V, and collision energy: 10 V. An information dependent acquisition (IDA) method was used in which the five most intense mass peaks were fragmented. MS/MS experiments were run with a collision energy of 40 V and collision energy spread of 15 V. Nitrogen was used as the drying, nebulizing, and collision gas.

### 2.5. Preparation of the Methanolic Extract

The methanol extraction step was performed following a method described by Barros et al. [14], slightly modified. Briefly, five grams of powdered mushrooms were extracted with 100 mL of methanol by shaking incubator (IST-3075R, Jeio Tech, Daejeon, Korea) at 25 °C at 200 rpm for six h. The supernatant was filtered through filter paper (Whatman No.4). The residue was extracted again using the same method described earlier. The combined methanolic extracts were evaporated at 40 °C to dryness using a rotary vacuum evaporator (N-1300E, Eyela, Tokyo, Japan) and were weighed. Then, the obtained extracts were freeze-dried and re-dissolved in methanol at the concentration of 10 mg/mL for further use. The extraction yield was calculated according to the following formula: extraction yield (%) = methanolic extract weight after solvent evaporation/sample weight of freeze-dried mushroom × 100.

### 2.6. Determination of Bioactive Compounds

The total phenolic content (TPC) was determined in accordance with colorimetric Folin–Ciocalteu assay with minor changes [15]. The absorbance (760 nm) was read in a multi-mode microplate reader. The phenolic content in the methanolic extract of *L. edodes* was calculated based on the calibration curve of gallic acid and expressed as milligram of gallic acid equivalents (GAE) per gram of dry methanolic extracts (D.E.).

The total flavonoid content (TFC) was performed according to the Davis method described by Huang et al. [16]. The absorbance was read at 420 nm in a spectrophotometer. The results were expressed using a calibration curve in mg of rutin equivalents (RE) per gram of dry methanolic extracts.

The tannin content was determined using a slightly modified vanillin-HCl method [17]. The absorbance was read at 500 nm in a spectrometer, and the results were expressed using a calibration curve in mg of catechin equivalents (CE) per gram of dry methanolic extracts.

The spectrophotometric measurements were performed by a multi-mode microplate reader (Synergy H1, BioTek Instruments, Winooski, VT, USA).

### 2.7. Determination of Antioxidant Capacity

The DPPH free radical scavenging activity of the methanolic extract of L. edodes was carried out as described with some modification [15]. The absorbance was read at a wavelength of 517 nm using a spectrophotometer. The DPPH radical-scavenging activity (RSA) was calculated by the following equation: % RSA = [(Acontrol-Asample)/Acontrol] × 100, where Acontrol is the absorbance of the control and Asample is absorbance of the tested sample. The results were expressed in half-minimal inhibitory concentration (IC_50_) calculated by comparing the RSA graph against the methanolic extract concentrations (2, 1, 0.5 0.25, and 0.125 mg/mL).

The ABTS free radical scavenging activity was determined following the method modified by the authors [18], and the absorbance of the final reaction mixture was read at 734 nm against an appropriate blank. The ABTS RSA of the methanolic extract was calculated by the following equation: % RSA = [1 − Asample/Acontrol] × 100. The half-minimal inhibitory concentration (IC_50_) was calculated from the graph of RSA percentage against the methanolic extract concentrations (10, 5, 2.5, 1.25, and 0.625 mg/mL).

The reducing antioxidant power was determined using the ferricyanide/Prussian blue method as described with slight modification [15]. The measurement of the absorbance was performed at 690 nm using a microplate reader. A higher absorbance indicates a robust reducing power. The extract concentration providing 0.5 of absorbance (IC_50_) was calculated from the graph of absorbance against the methanolic extract concentrations (10, 5, 2.5, 1.25, and 0.625 mg/mL) of *L. edodes*.

### 2.8. Statistical Analysis

Extraction yield, bioactive compounds, and antioxidant capacity were performed in triplicate, and all data were presented as mean ± standard deviations. The statistical significance of the data was analyzed by the Mann–Whitney U test using SPSS version 20.0 program (SPSS Inc., Chicago, IL, USA).

Multivariate statistical analyses were performed with a unit variance scale using SIMCA-P+ software, version 16.0 (Umetrics, Umeå, Sweden).

## 3. Results and Discussion

### 3.1. Metabolite Profiling Using ^1^H NMR

**Figure 1:**
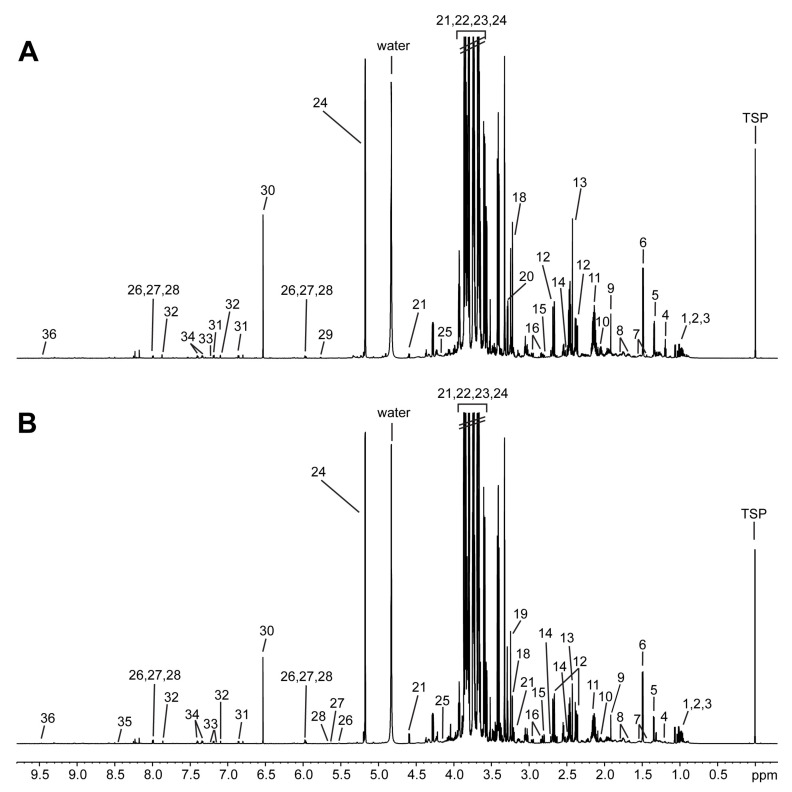
Representative ^1^H NMR spectra of *L. edodes* extracts cultivated on (**A**) log bed and (**B**) sawdust media. (1, isoleucine; 2, leucine; 3, valine; 4, ethanol; 5, threonine; 6, alanine; 7, lysine; 8, arginine; 9, acetate; 10, glutamate; 11, glutamine; 12, malate; 13, succinate; 14, citrate; 15, aspartate; 16, asparagine; 17, ornithine; 18, choline; 19, sn-Glycero-3-phosphocholine; 20, trimethylamine N-oxide; 21, glucose; 22, arbinitol; 23, mannitol; 24, trehalose; 25, myo-Inositol; 26, UDP-N-Acetylglucosamine; 27, UDP-glucose; 28, UDP-galactose; 29, uracil; 30, fumarate; 31, tyrosine; 32, histidine; 33, tryptophan; 34, phenylalanine; 35, formate; 36, NAD+).

To determine metabolic differences in the fruiting bodies of mushrooms cultivated on logs versus sawdust, we analyzed *L. edodes* extracts using ^1^H NMR. Representative 800 MHz ^1^H NMR spectra of *L. edodes* mushrooms grown on logs versus sawdust are shown in Figure 1.

The dominant signal in each ^1^H NMR spectrum represents carbohydrates (3.0–4.5 ppm), and all samples regardless of culture conditions showed similar spectra suggesting similar metabolic profiles. However, further analysis showed that the amounts of many metabolites were distinctly different between the two groups. Spectral resonances of metabolites were assigned based on comparison with the chemical shifts of standard compounds using the 800 MHz library from Chenomx NMR Suite Version 8.6, 2D NMR spectra (Figure S1), and spiking experiments. The analysis of *L. edodes* fruiting body extracts by ^1^H NMR revealed 36 essential primary metabolites including amino acids, organic acids, and sugars that were quantified using the Chenomx NMR Suite (Table 1).

This program uses the concentration of a known reference signal (in this case TSP) to determine the concentration of individual metabolites.

**Figure 2:**
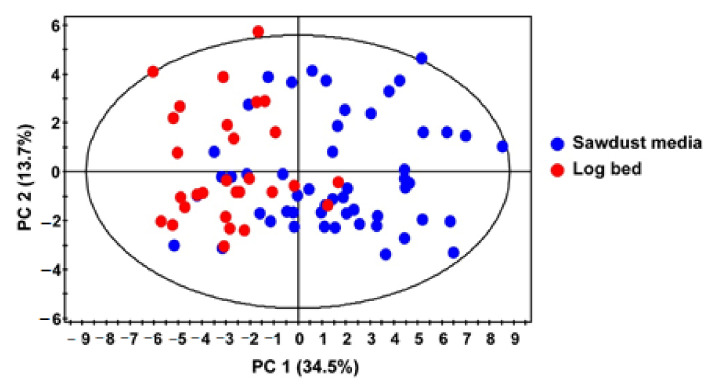
PCA score plot of quantified metabolites from ^1^H NMR analysis in *L. edodes* extracts.

Principal components analysis (PCA) was performed on quantified metabolites of *L. edodes* mushroom extracts to identify primary metabolite variations that correlated with the cultivation method. PCA is an unsupervised clustering method requiring no prior knowledge of the data set that reduces the dimensionality of multivariate data while maintaining most of the variance [19]. The PCA score plot was established with four components and showed a clear separation between log-grown and sawdust-grown groups with high goodness-of-fit (R^2^ = 0.644) and predictability (Q^2^ = 0.432). The PCA score plot showed differences in metabolites along the first principal component (Figure 2) demonstrating that the cultivation method affects the primary metabolite content of *L. edodes.*

The Mann–Whitney *U* test showed that 23 of the 36 identified metabolites had statistically significant differences in concentration between *L. edodes* mushrooms grown on different substrates. Overall, the primary metabolites that changed were significantly decreased in *L. edodes* grown on logs versus sawdust.

Most amino acids that showed significant differences in concentration had higher concentrations in *L. edodes* mushrooms grown on sawdust compared to logs, which was similar to the results of a previous study that measured total free amino acids [13]. Amino acids play a crucial role in cellular metabolism and protein synthesis, and mushrooms have proven to be good sources of almost all essential amino acids, comparable to common vegetables [5]. Amino acids are also the major umami component in *L. edodes* and are responsible for various physiological and pharmacological characteristics [20]. The amino acid content of mushrooms is related to maturity, quality grade, preservation methods, and post-harvest storage [21,22].

Malate was the major organic acid in *L. edodes*, which is consistent with previous results [23]. The only organic acid that showed a difference in concentration between the two groups was citrate, indicating that the cultivation environment of *L. edodes* had little or no effect on the concentration of organic acids.

Mannitol, trehalose, and arabinitol are the main soluble sugars in *L. edodes* [23,24], and our study found that these sugars were present at high concentrations in mushrooms grown on either substrate. Only mannitol showed a significant difference in concentration between the two groups with higher levels in *L. edodes* harvested from sawdust than from logs.

Nucleotide sugars are universal sugar donors that are important in the synthesis of polysaccharides, glycoproteins, proteoglycans, glycolipids, and glycosylated secondary metabolites [25]. We identified and quantified three nucleotide sugars (UDP-N-acetylglucosamine, UDP-glucose, and UDP-galactose) and determined that they were all present at lower concentrations in *L. edodes* grown on logs compared to those grown on sawdust.

### 3.2. Metabolite Profiling Using UPLC-QTOF-MS

Primary metabolites including amino acids, organic acids, and sugars were detected by ^1^H NMR analysis of *L. edodes* extracts. However, secondary metabolites such as phenolic acids and flavonoids were not present in the ^1^H NMR data; therefore, UPLC-QTOF-MS metabolic profiling was performed (Figure S2). Metabolites were identified based on their retention times, mass values, and MS/MS spectral data, and 17 metabolites were tentatively identified based on exact mass measurements of molecular ions and fragment ions (Table 2).

The concentrations of 15 metabolites, including phenolic compounds, were significantly different between the two mushroom groups based on the Mann–Whitney *U* test. Phenolic compounds, which share an aromatic ring with one or more hydroxyl groups [26], are metabolites derived from secondary pathways in plants.

Cinnamic acid, which is in the phenylpropanoid pathway and has strong reactive oxygen scavenging activity [27], was increased in *L. edodes* cultivated on logs. Benzoic acid and its derivatives, including 3,4-dimethoxybenzoic acid, and syringic acid were also significantly higher in *L. edodes* cultivated on logs compared to sawdust, but vanillic acid concentrations were similar between the two groups. In contrast, coumaric acid and ferulic acid were higher in *L. edodes* grown on sawdust. These compounds have antioxidant, anti-inflammatory, and potential anticancer activities [28,29].

Catechin, known as an antioxidant with anticancer activity, was markedly increased in *L. edodes* cultivated on logs (Table 2) [30]. Apigenin and glycitein were significantly higher in *L. edodes* grown on sawdust. Apigenin, one of several natural flavones with many biological functions, is found in many plants. Riboflavin (vitamin B2) was significantly increased in *L. edodes* cultivated on logs. It is a micronutrient that is required for antibody production and for regulating human growth and reproduction. The levels of niacinamide (vitamin B complex) and ascorbic acid (vitamin C) were also higher in *L. edodes* grown on sawdust compared to growth on logs.

### 3.3. Extraction Yield and Analysis of Bioactive Components

There was no significant difference in yield for methanol extracts of samples grown on logs (24.94%) compared to sawdust (25.08%) (Table 3).

Our results fell within the range for yield for commercial methanol extracts of *L. edodes* (15.9–36.1%) [31,32,33].

Phenolic compounds are strongly correlated with the antioxidant capacity of plant materials [34]; therefore, we measured the TPC of mushroom extracts. The TPC for methanolic extracts of *L. edodes* grown on logs (5.06 mg GAE/g D.E.) was higher than for *L. edodes* grown on sawdust (4.86 mg GAE/g D.E.) indicating that the cultivation conditions influence the phenolic compound content of mushrooms. Wild mushrooms have higher levels of phenolic compounds than cultivated species [15,17,35]; thus, our results suggest that logs may provide an environment similar to the wild environment that supports the growth of *L. edodes* and that this environment could explain the higher phenol content.

Methanolic extracts of log-cultivated *L. edodes* had significantly higher TFC (1.35 ± 0.43 mg RE/g D.E.) than the extract from sawdust-grown mushrooms (1.20 ± 0.53 mg RE/g D.E.). Since flavonoids and phenolics contribute to the antioxidant effect of medicinal plants [36], *L. edodes* cultivated on logs with high flavonoid content would have more antioxidants than *L. edodes* cultivated on sawdust.

The tannin content in methanolic extracts of samples from both mushroom groups was low (0.62 mg CE/g D.E.) with no significant difference between the groups.

### 3.4. Determination of Antioxidant Capacity

The antioxidant capacity of methanolic extracts of *L. edodes* was determined using DPPH free radical scavenging, ABTS free radical scavenging, and the reducing power. The antioxidant capacity of *L. edodes* grown on logs versus sawdust was expressed as IC_50_ values (Table 4).

The free radical scavenging activity, based on the DPPH assay, for *L. edodes* cultivated on logs and sawdust, resulted in IC_50_ values of 0.88 ± 0.17 mg/mL and 0.94 ± 0.17 mg/mL, respectively. These values were lower than the values reported for the inner cap, stipes, fruiting body of *L. edodes* (0.169, 0.213, and 0.141 mg/mL, respectively) [37] and sequential extracts of *L. edodes* (0.15–0.17 mg/mL) [38]. The RSA of the methanolic extracts using the ABTS assay was calculated as a percentage increase with the concentration. The *L. edodes* cultivated on logs showed greater activity (IC_50_ = 1.90 mg/mL) compared to those cultivated on sawdust (IC_50_ = 2.38 mg/mL). The reducing power was determined using the ferricyanide/Prussian blue assay based on the reduction of ferric iron [17]. The IC_50_ values based on the reducing ability of methanolic extracts of *L. edodes* cultivated on logs or sawdust (2.33 and 2.92 mg/mL, respectively) were similar to a previous report for mushrooms grown in a solid medium (2.26 mg/mL) [39].

*L. edodes* cultivated on logs had higher free radical scavenging activity than *L. edodes* grown on sawdust. Similarly, the TPC and TFC in mushrooms grown on logs were higher than for mushrooms grown on sawdust. Thus, higher antioxidant capacity correlated well with greater total phenolics and flavonoids, and our results were similar.

## 4. Conclusions

This study is the first to report the metabolic profile, bioactive compounds, and antioxidant capacity of *L. edodes* fruiting bodies produced on logs versus sawdust substrates. Metabolic profiling using ^1^H NMR and UPLC-QTOF-MS analysis revealed that the concentration of primary and secondary metabolites in *L. edodes* mushrooms was significantly different in *L. edodes* grown on different substrates. Most of the primary metabolites were present at higher concentrations in *L. edodes* cultivated on sawdust, which may be the result of greater primary metabolism due to the faster growth of *L. edodes* cultivated on sawdust compared to logs. In contrast, *L. edodes* cultivated on logs had increased bioactive compounds and antioxidant capacity compared to sawdust, and growth on logs was associated with higher concentrations of TPC and TFC. Further experiments in vitro and in vivo will be needed to determine the possible benefits of an increased antioxidant content in *L. edodes*. Our results provide information on key differences between log and sawdust cultivation for *L. edodes* that can be used to decide which method is advantageous for improving the nutritional and/or medicinal benefits of *L. edodes* mushrooms.

## Figures and Tables

**Table 1:** Metabolites detected by ^1^H NMR in *Lentinula edodes* extracts.

No.	Metabolite	Log Bed (μM, n = 29)	Sawdust Media (μM, n = 52)	x-Fold Change
				Log Bed/Sawdust Media
1	Acetate	397.88 ± 693.76	675.71 ± 1225.33	0.59
2	Formate	6.44 ± 2.25	6.52 ± 2.03	0.99
3	Citrate	527.57 ± 211.06	392.08 ± 103.01	1.35 **
4	Fumarate	1024.32 ± 252.79	1112.18 ± 225.77	0.92
5	Malate	4782.12 ± 893.68	5052.55 ± 886.63	0.95
6	Succinate	365.91 ± 181.96	300.12 ± 162.8	1.22
7	Alanine	1208.7 ± 487.68	1174.01 ± 467.17	1.03
8	Arginine	442.31 ± 226.7	641.1 ± 267.12	0.69 **
9	Asparagine	499.69 ± 176.29	588.61 ± 187.99	0.85 *
10	Aspartate	618.73 ± 434.3	707.73 ± 407.19	0.87
11	Glutamate	1005.86 ± 331.19	1267.12 ± 440.44	0.79 **
12	Glutamine	5447.25 ± 1941.88	6693.75 ± 2364.08	0.81 *
13	Histidine	162.39 ± 56.05	197.05 ± 68.67	0.82 *
14	Isoleucine	132.63 ± 51.58	225.35 ± 102.73	0.59 ***
15	Leucine	189.6 ± 68.7	322.53 ± 139.72	0.59 ***
16	Lysine	453.17 ± 146.7	729.61 ± 217.9	0.62 ***
17	Ornithine	635.24 ± 280.67	1054.49 ± 411.71	0.60 ***
18	Phenylalanine	88.08 ± 25.01	134.59 ± 43.22	0.65 ***
19	Threonine	488.2 ± 132.49	655.21 ± 176.73	0.75 ***
20	Tryptophan	35.15 ± 15.18	51.49 ± 18.8	0.68 ***
21	Tyrosine	88.52 ± 30.03	173.57 ± 86.12	0.51 ***
22	Valine	257.18 ± 89.27	442.15 ± 179.74	0.58 ***
23	Arabinitol	11,832.42 ± 5501.65	11,989.78 ± 5200.2	0.99
24	Mannitol	18,828.69 ± 3851.1	26,495.17 ± 6170.15	0.71 ***
25	Trehalose	10,132.53 ± 2783.68	8688.03 ± 3165.51	1.17
26	Glucose	463.2 ± 753.62	370.91 ± 757.63	1.25
27	myo-Inositol	208.54 ± 59.41	258.13 ± 149.75	0.81
28	UDP-N-Acetylglucosamine	63.28 ± 11.53	112.86 ± 23.95	0.56 ***
29	UDP-galactose	19.8 ± 3.5	28.25 ± 5.5	0.70 ***
30	UDP-glucose	63.16 ± 11.03	97.21 ± 16.49	0.65 ***
31	Ethanol	186.21 ± 270.16	91.77 ± 148.89	2.03 *
32	NAD+	35.23 ± 5.38	46.46 ± 8.98	0.76 ***
33	Uracil	16.65 ± 15.27	11.78 ± 12.73	1.41
34	Choline	504.11 ± 231.99	231.63 ± 138.76	2.18 ***
35	sn-Glycero-3-phosphocholine	383.91 ± 125.94	816.89 ± 310.72	0.47 ***
36	Trimethylamine N-oxide	183.94 ± 37.73	173.6 ± 60.96	1.06

The results are presented as mean ± SD. * *p* < 0.05, ** *p* < 0.01, and *** *p* < 0.001 indicate significant differences between samples cultivated on log bed and sawdust media.

**Table 2:** Metabolites detected by UPLC-QTOF-MS in *Lentinula edodes* extracts.

No.	Proposed Identity	Rt (min)	Ionization Mode	Precursor Ions (*m*/*z*)		Difference (ppm)	Molecular Formula	x-Fold Change
				Theoretical	Observed			Log Bed/Sawdust Media
1	3,4-Dimethoxybenzoic acid	3.69	−	181.0501	181.0502	0.64	C_9_H_10_O_4_	1.24 ***
2	Apigenin	6.8	−	269.045	269.0463	4.83	C_15_H_10_O_5_	0.74 *
3	Benzoic acid	0.75	−	121.029	121.0284	4.59	C_7_H_6_O_2_	3.17 ***
4	Caffeic acid	3.86	−	179.0344	179.0344	0.2	C_9_H_8_O_4_	0.33
5	Catechin	3.27	−	289.0712	289.0718	2.02	C_15_H_14_O_6_	7.2 **
6	Cinnamic acid	5.72	−	147.0446	147.0446	0.03	C_9_H_8_O_2_	1.31 ***
7	Coumaric acid	4.35	+	165.0552	165.0538	8.3	C_9_H_8_O_3_	0.15 ***
8	Ferulic acid	4.59	−	193.0501	193.0517	8.37	C_10_H_10_O_4_	0.40 *
9	Gallic acid	2.49	−	169.0137	169.012	10.06	C_7_H_6_O_5_	1.06 ***
10	Glycitein	5.58	−	283.0607	283.0608	0.53	C_16_H_12_O_5_	0.09 ***
11	L-ascorbic acid	0.81	−	175.0243	175.0225	10.08	C_6_H_8_O_6_	0.23***
12	Niacinamide	2	+	123.0558	123.0546	10.06	C_6_H_6_N_2_O	0.14 *
13	Riboflavin	3.85	+,−	377.1461	377.1457	1.09	C_17_H_20_N_4_O_6_	4.28 ***
14	Salicylic acid	4.5	−	137.0239	137.0228	7.81	C_7_H_6_O_3_	2.92
15	Shikimic acid	0.8	−	173.045	173.0433	9.82	C_7_H_10_O_5_	1.10 ***
16	Syringic acid	2.57	−	197.045	197.0449	0.51	C_9_H_10_O_5_	1.34 ***
17	Vanillic acid	3.44	−	167.0344	167.0349	2.78	C_8_H_8_O_4_	0.34 **

* *p* < 0.05, ** *p* < 0.01, and *** *p* < 0.001 indicate significant differences between samples cultivated on log bed and sawdust media.

**Table 3:** Bioactive components of the methanolic extract in *Lentinula edodes* cultivated on log bed and sawdust media.

Bioactive Compounds	*L. edodes*		
	Log Bed (n = 29)	Sawdust Media (n = 52)	*p* Value
Extraction yield (%)	24.94 ± 4.48	25.08 ± 3.36	0.420 ^ns^
Total phenolic content (mg GAE/g dry extract)	5.06 ± 0.62	4.86 ± 0.68	0.017 *
Total flavonoid content (mg RE/g dry extract)	1.35 ± 0.43	1.20 ± 0.53	0.000 ***
Total tannin content (mg CE/g dry extract)	0.62 ± 0.22	0.62 ± 0.26	0.583 ^ns^

The results are presented as mean ± SD of triplicates. ^ns^ *p* > 0.05; * *p* < 0.05 and *** *p* < 0.001 indicate significant differences between samples cultivated on log bed and sawdust media.

**Table 4:** IC_50_ (mg/mL) properties of the methanolic extracts in *Lentinula edodes* cultivated on log bed and sawdust media.

Assays	*L. edodes*		
	Log Bed (n = 29)	Sawdust Media (n = 52)	*p* Value
DPPH radical-scavenging activity	0.88 ± 0.17	0.94 ± 0.17	0.012 *
ABTS radical-scavenging activity	1.90 ± 0.42	2.38 ± 0.61	0.000 ***
Reducing power activity	2.33 ± 0.58	2.92 ± 0.53	0.000 ***

The results are presented as mean ± SD (n = 3) of triplicates. * *p* < 0.05 and *** *p* < 0.001 indicate significant differences between samples cultivated on log bed and sawdust media.

## Supplementary Materials

The following are available online at https://www.mdpi.com/article/10.3390/biom11111654/s1, Figure S1: Expansion of representative 2D NMR spectra (TOCSY) of *L. edodes* extracts, Figure S2: The spectra of UPLC-QTOF-MS from *L. edodes* extracts, Table S1: Sample list of *Lentinula edodes*.
