# Supplementary material for: Metabolic Profiles, Bioactive Compounds, and Antioxidant Capacity in Lentinula edodes Cultivated on Log versus Sawdust Substrates
Source: Biomolecules. 2021 Nov 8;11(11):1654. doi: 10.3390/biom11111654 (PMC8615513; doi:10.3390/biom11111654)
Supplement: Supplementary file 1 [file biomolecules-11-01654-s001.zip › biomolecules-1430827-supplementary.pdf]

Metabolic Profiles, Bioactive Compounds, and Antioxidant Capacity in *Lentinula edodes* Cultivated on Log versus Sawdust Substrates

Miso Nam, Ji Yeon Choi, Min-Sun Kim

Food Analysis Research Center, Korea Food Research Institute, Wanju 55365, Korea

Supplementary Table 1. Sample list of *Lentinula edodes*

Cultivation condition	Collection strains	Number of samples
Log bed	Mori 290	5
	Mori 436	1
	Sanjo 501	1
	Sanjo 502	3
	Yujiro	19
Sawdust media	L808	24
	Sanjo 701	9
	Sanjo 715	4
	Chamaram	10
	Chujae 2ho	5

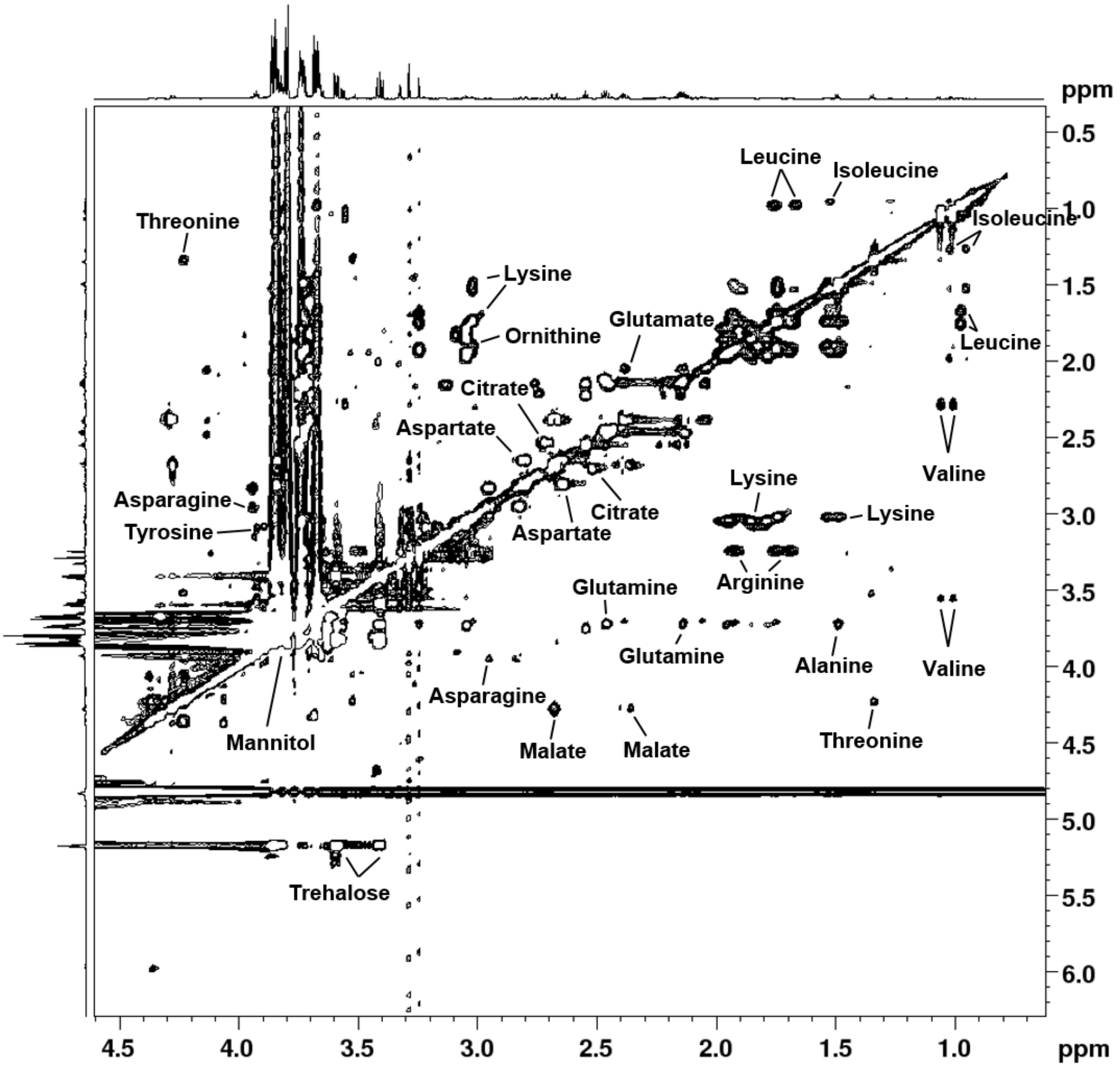

Supplementary Figure 1. Expansion of representative 2D NMR spectra (TOCSY) of *L. edodes* extracts.

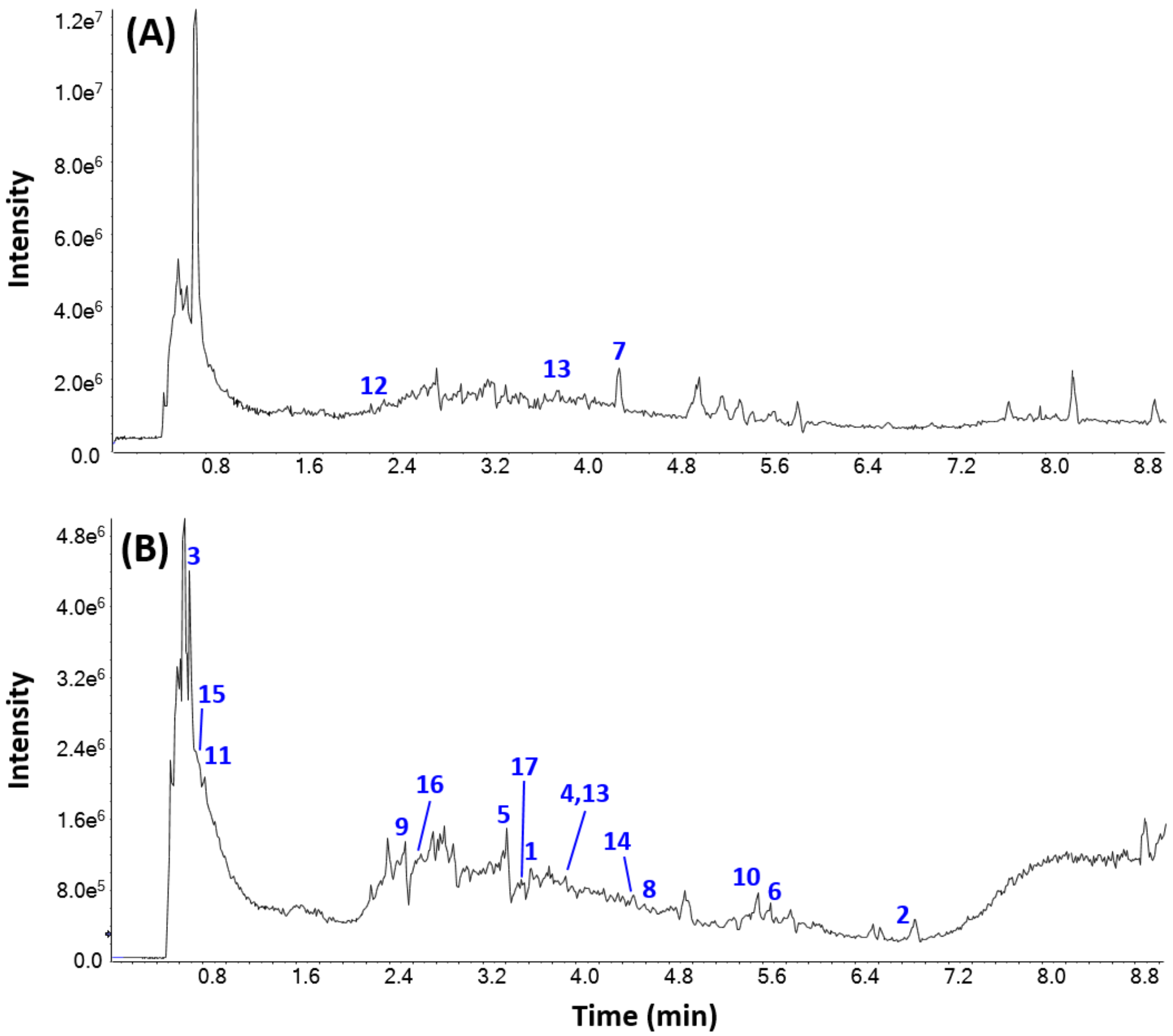

Supplementary Figure 2. The spectra of UPLC-QTOF-MS in (A) ESI-positive and (B) ESI-negative modes from *L. edodes* extracts.

Peak identification: 1. 3,4-Dimethoxybenzoic acid; 2. Apigenin; 3. Benzoic acid; 4. Caffeic acid; 5. Catechin; 6. Cinnamic acid; 7. Coumaric acid; 8. Ferulic acid; 9. Gallic acid; 10. Glycitein; 11. L-ascorbic acid; 12. Niacinamide; 13. Riboflavin; 14. Salicylic acid; 15. Shikimic acid; 16. Syringic acid; 17. Vanillic acid.
